# Phloem unloading in cultivated melon fruits follows an apoplasmic pathway during enlargement and ripening

**DOI:** 10.1093/hr/uhad123

**Published:** 2023-07-04

**Authors:** Yixuan Zhou, Kexin Li, Suying Wen, Dong Yang, Jun Gao, Ziwei Wang, Peilu Zhu, Zhilong Bie, Jintao Cheng

**Affiliations:** National Key Laboratory for Germplasm Innovation and Utilization for Fruit and Vegetable Horticultural Crops, College of Horticulture and Forestry Sciences, Huazhong Agricultural University, Wuhan 430070, Hubei Province, China; National Key Laboratory for Germplasm Innovation and Utilization for Fruit and Vegetable Horticultural Crops, College of Horticulture and Forestry Sciences, Huazhong Agricultural University, Wuhan 430070, Hubei Province, China; National Key Laboratory for Germplasm Innovation and Utilization for Fruit and Vegetable Horticultural Crops, College of Horticulture and Forestry Sciences, Huazhong Agricultural University, Wuhan 430070, Hubei Province, China; National Key Laboratory for Germplasm Innovation and Utilization for Fruit and Vegetable Horticultural Crops, College of Horticulture and Forestry Sciences, Huazhong Agricultural University, Wuhan 430070, Hubei Province, China; National Key Laboratory for Germplasm Innovation and Utilization for Fruit and Vegetable Horticultural Crops, College of Horticulture and Forestry Sciences, Huazhong Agricultural University, Wuhan 430070, Hubei Province, China; National Key Laboratory for Germplasm Innovation and Utilization for Fruit and Vegetable Horticultural Crops, College of Horticulture and Forestry Sciences, Huazhong Agricultural University, Wuhan 430070, Hubei Province, China; National Key Laboratory for Germplasm Innovation and Utilization for Fruit and Vegetable Horticultural Crops, College of Horticulture and Forestry Sciences, Huazhong Agricultural University, Wuhan 430070, Hubei Province, China; National Key Laboratory for Germplasm Innovation and Utilization for Fruit and Vegetable Horticultural Crops, College of Horticulture and Forestry Sciences, Huazhong Agricultural University, Wuhan 430070, Hubei Province, China; National Key Laboratory for Germplasm Innovation and Utilization for Fruit and Vegetable Horticultural Crops, College of Horticulture and Forestry Sciences, Huazhong Agricultural University, Wuhan 430070, Hubei Province, China

## Abstract

Melon (*Cucumis melo* L.) has a long history of cultivation worldwide. During cultivation, domestication, and selection breeding, the sugar content of mature melon fruits has been significantly increased. Compared with unsweet melon and wild melon, rapid sucrose accumulation can occur in the middle and late stages of sweet melon fruit development. The phloem unloading pathway during the evolution and development of melon fruit has not been identified and analyzed. In this study, the phloem unloading pathway and the function of related sugar transporters in cultivated and wild melon fruits were analyzed by CFDA [5(6)-carbofluorescein diacetate] and esculin tracing, cytological pathway observation, qRT–PCR, and gene function analysis, etc. Results show that the phloem unloading pathway of wild melon fruit is largely symplastic, whereas the phloem unloading pathway of cultivated melon fruit shifts from symplastic to apoplasmic during development. According to a fruit grafting experiment, the fruit sink accumulates sugars independently. Correlation analysis showed that the expression amounts of several sucrose transporter genes were positively correlated with the sucrose content of melon fruit. Furthermore, CmSWEET10 was proved to be a sucrose transporter located on the plasma membrane of the phloem and highly expressed in the premature stage of sweet melon fruits, which means it may be involved in phloem apoplast unloading and sucrose accumulation in sweet melon fruits. Finally, we summarize a functional model of related enzymes and sugar transporters involved in the apoplast unloading of sweet melon fruits during enlargement and sucrose accumulation.

## Introduction

Sugar is transported from sources to sinks through three steps: loading into the phloem of source leaves, long distance flow within the phloem, and unloading to sink organs. During the past decade, phloem transport research has focused on phloem loading and unloading [1]. Phloem unloading of photoassimilates in fruits is a key factor in determining yield and quality [2]. In most sink tissues, such as sink leaves, roots, and bud growth points, the unloading of assimilates is primarily achieved by symplastic unloading [1]. However, during the development of fruits that accumulate high concentrations of sugar, such as pear [3], apples [4], and grapes [5], assimilates are widely unloaded through the apoplasmic pathway to overcome the inverse concentration gradient between the source and the sink [6]. The apoplasmic unloading pathway of sugar in sink organs will undergo three steps of transmembrane transport. Sucrose is first transported from the sieve element–companion cell (SE–CC) complex to the apoplasmic space through the SWEET (Sugars Will Eventually be Exported Transporter) [7], and then the sucrose in the extracellular space is further transported into the parenchyma cells (PCs) by sucrose transporter (SUT) [8] or it is hydrolyzed into hexose by cell wall invertase (CwIN) [9] and then transported to PCs by hexose transporter (HT/STP) [10]. Finally, the excess sucrose will be transported into the vacuole for storage through the tonoplast sugar transporters (TST) [11], such as in sugar beet taproots [12] and fruit flesh (e.g. apple [13], strawberry [14], peach [15], and apricot [16]).

The melon (*Cucumis melo* L.) is one of the most widely grown crops of the Cucurbitaceae family and is consumed worldwide [17]. The development of melon fruit includes four typical stages: young fruit stage [~0–10 days after pollination, (DAP)], expanding stage (~10–20 DAP), premature stage (~20–30 DAP), and mature stage (after 30 DAP) [11]. The sweet melon fruit shows rapid sucrose accumulation during the premature stage, which is different from unsweet and wild melon fruit [18, 19]. In the Cucurbitaceae, raffinose family oligosaccharides (RFOs) are the predominant sugars transported between source and sink [20, 21]. RFOs should be hydrolyzed into galactose and sucrose by α-galactosidase (neutral-alkaline α-galactosidase and acid α-galactosidase, NAG and AAG) [22–24] after reaching the phloem of peduncle and fruit. It has been reported that the phloem unloading pathway of watermelon and cucumber fruits follows the apoplasmic pathway [25]. Several sugar transporters (CsSWEET7a, CsSWEET10, CsHT3, and CsSUT4) have been found to play important roles in phloem sugar unloading of cucumber [25–28]. Similarly, several key enzymes and sugar transporters (ClAGA2, ClVST1, ClSWEET3, and ClTST2) involved in sugar unloading and accumulation in watermelon fruit have been found through forward genetics [29–31]. During the evolution of watermelon from an unsweet ancestor to sweet watermelon, ClAGA2, ClSWEET3, and ClTST2 were selected to evolve, thereby promoting carbohydrate allocation and storage [31]. Like watermelon fruit, melon fruit can also accumulate a large amount of sugar. As a fruit with a long cultivation history, melon has produced several varieties during domestication [32], with the most striking difference among varieties regarding sugar content. However, the phloem unloading pathway and sugar accumulation mechanism of melon fruit have not been systematically studied.

Here, we found that the phloem unloading of wild melon fruit is mainly symplastic throughout the fruit development process, whereas the phloem unloading of cultivated melon fruit shifts from symplasmic to apoplasmic during fruit development. Moreover, we depicted the spatiotemporal expression patterns of the genes involved in sugar transport and metabolism in melon fruit. We identified several sugar-correlated genes through correlation analysis between transcript abundance and sugar content in six varieties with different sugar levels. Finally, we determined the classification, substrate specificity, subcellular localization, and tissue localization of CmSWEETs and summarize a functional model of related proteins.

## Results

### Phloem unloading pathway of cultivated melon shift from symplasmic to apoplasmic during fruit development

The melon fruit is developed from an ovary, and it usually accumulates high levels of sucrose in the flesh after ripening. In investigating why melon fruit can accumulate high levels of sugar and how sugars unload in fruit, we loaded carboxyfluorescein (CF) diacetate (DA) into the melon fruit. CF is widely used as a fluorescent marker to determine photoassimilate transport and unloading [4, 6, 25, 30]. The non-fluorescent and membrane-permeable CFDA is degraded to CF, a membrane-impermeable fluorescent dye, when it is loaded into cells.

**Figure 1:**
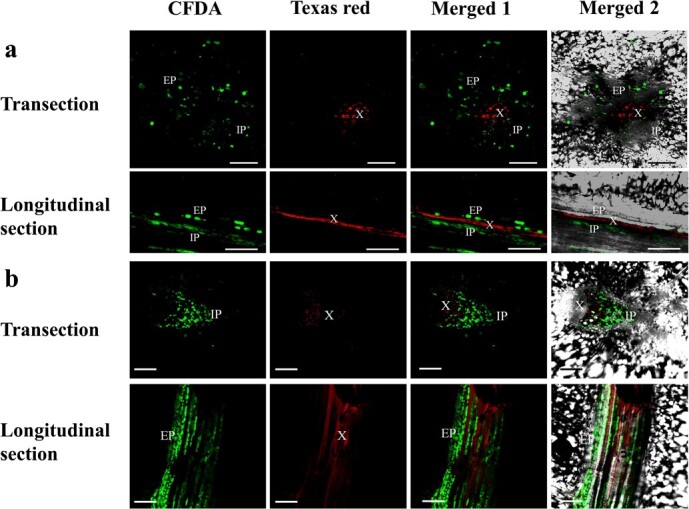
CLSM imaging of CF unloading in premature stage of YL (**a**, high sugar content cultivated) and BCG (**b**, low sugar content cultivated) melon fruit, observed by monitoring the movement of CF, a symplasmic dye. CF fluorescence was detected in fruit after 24 hours of feeding with CFDA. The picture shows the main vascular bundle of the fruit, as shown in Fig. 3a and b. CF fluorescence can be observed only in the external phloem (EP) and internal phloem (IP) cells. Red fluorescence (Texas red) shows the xylem (X). Scale bars = 200 μm.

**Figure 2:**
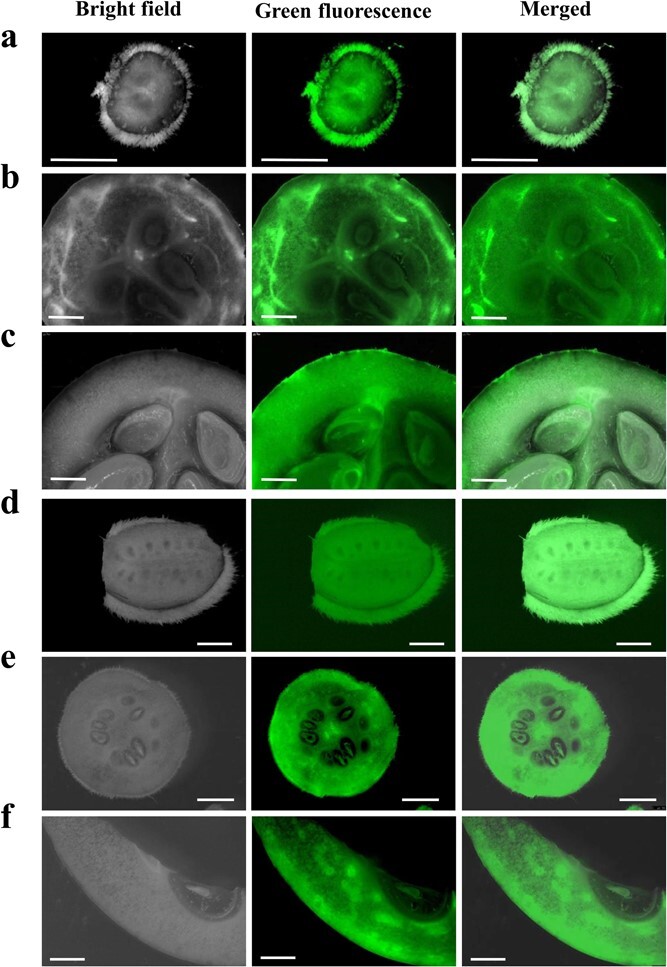
CF transport imaging in wild varieties melon fruits of MPG (**a**–**c**) and JKY (**d**–**f**). **a** and **d**, 0 DAP fruits; **b** and **e**, 5 DAP fruits; **c** and **f** represent premature fruits. Scale bars = 2 mm.

Here, different developmental stages of fruits from eight different melon varieties (sweet melon: YL, XMS, YJS, YN, M1-43; unsweet melon: BCG; wild melon: JKY, MPG) were used (Supplementary Data Fig. S1, Supplementary Data Table S1). The results showed that the CF was released from the phloem strands and spread all over the fruit tissues during the young fruit stage (~0–5 DAP) of cultivated melons (Supplementary Data Fig. S2a, d, and g). However, the CF was confined to the phloem zones of the vascular bundles without apparent spread to the surrounding pulp parenchyma cells at the expanding and premature stages (~5–30 DAP) (Fig. 1, Supplementary Data Fig. S2b, c, e, f, and h), even when the samples were collected 48 hours after the application of CF (data not shown), which indicates that phloem unloading in these fruits follows an apoplasmic pathway. These results indicated that the phloem unloading pathway of cultivated melon fruit shifts from symplasmic to apoplasmic during development. Interestingly, we observed different results in wild melon fruits (JKY and MPG). The CF spread across the fruit tissue throughout the developmental period of wild melon (Fig. 2, Supplementary Data Fig. S3), which indicated that phloem unloading in wild melon fruits mainly followed a symplastic pathway during fruit development (0–30 DAP).

In order to distinguish the phloem and xylem, a widely used tracer of xylem vessels, Texas red dextran (3 kDa), was also introduced into the main vein of melon fruit. Texas red fluorescence and CF fluorescence can be clearly distinguished without overlapping each other (Fig. 1), which indicates that the introduction of CF into melon fruit was effective, and the results of CF unloading were reliable.

**Figure 3:**
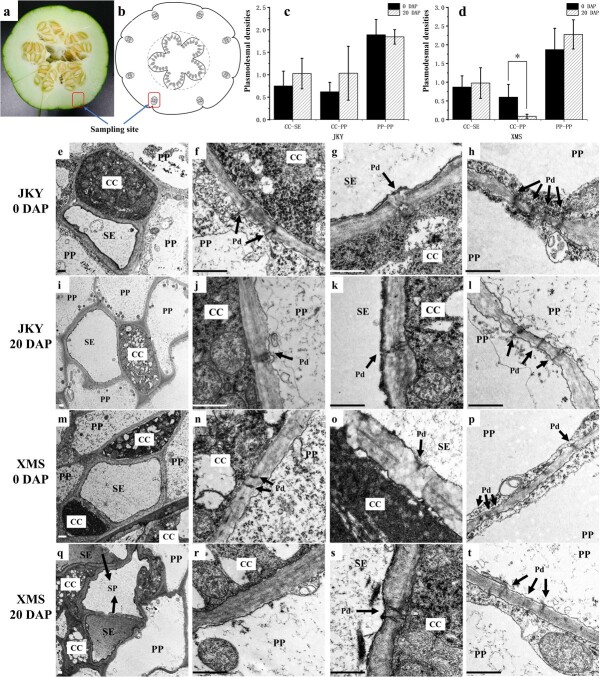
Phloem cell ultrastructure of melon fruit. **a**, **b** Photograph (**a**) and diagram (**b**) of transverse section of fruit of XMS melon, showing the site of sampling (indicated by blue arrow) and the vascular bundle distribution. **c**, **d** Plasmodesmata densities in different cell types in phloem of JKY (**c**) and XMS (**d**) fruit. Each value represents the mean ± standard deviation of three replications. ^*^Significant at *P* < .05 (*t*-test). **e**–**t** Ultrastructure of phloem (**e**, **i**, **m**, **q**), PP–CC (**f**, **j**, **m**, **r**), SE–CC (g, k, o, s), and PP–PP (**h**, **l**, **p**, **t**) in cultivated melon fruits (XMS) and wild melon fruits (JKY). Black arrow points to plasmodesmata and sieve plate. CC, companion cell; PP, phloem parenchyma cell; SE, sieve element; Pd, plasmodesmata; SP, sieve plate. Scale bars = 1 μm.

To assess the plasmodesmal connections between SE–CC complexes and surrounding cells more reliably, the ultrastructure of vascular bundles (Fig. 3a and b) of fruits in the young fruit stage (0 DAP) and premature stage (20 DAP) of cultivated melon (XMS) and wild melon (JKY) were observed, and the plasmodesmal densities were counted and compared (Fig. 3, Supplementary Data Fig S4). The results showed that there were abundant plasmodesmata between SE–CC and PP (phloem parenchyma cell)–PP in JKY (Fig. 3c, g, h, k, and l) and XMS (Fig. 3d, o, p, s, and t) fruits at both stages. Furthermore, plasmodesmata were found at the interface between the SE–CC complex and its adjacent parenchyma in fruits of both JKY (Fig. 3e and f) and XMS (Fig. 3m and n) at the young fruit stage. However, at the premature stage, plasmodesmata between the CC and its surrounding PPs could be easily observed in JKY fruits (Fig. 3i and j) but were rarely observed in XMS fruits (Fig. 3q and r). Further statistical analysis of the density of plasmodesmata revealed that the number of plasmodesmata between CC and PP in XMS fruit at premature stage (20 DAP) was significantly lower than that at young fruit stage (0 DAP) (Fig. 3d). In premature stage, the number of plasmodesmata between CC–PPs in XMS fruits was also significantly lower than that in JKY fruits (Supplementary Data Fig. S4).

**Figure 4:**
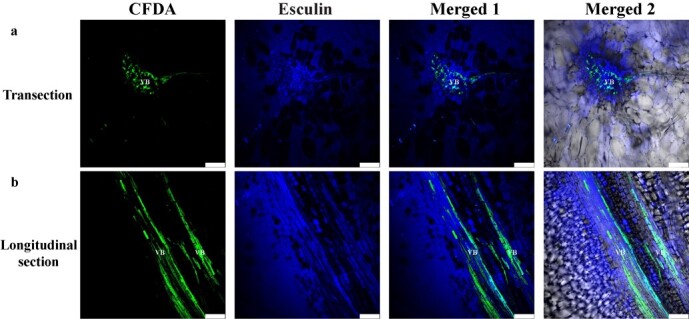
CLSM imaging of CF and esculin transport in premature stage of fruit (cv. XMS). The esculin probe was detected in both fruit vascular bundle and surrounding parenchymal cells. The CFDA was restricted to the phloem after >24 h of loading. VB, vascular bundle. Bars = 50 μm.

In order to prove whether sucrose transporters are involved in apoplast unloading, we introduced the fluorescent sucrose derivative esculin into melon fruit after CFDA labeling. In melon fruit, esculin entered the phloem vascular bundles and was unloaded from the phloem to surrounding parenchymal cells within 1 hour (Fig. 4), which indicates that sucrose transporters are involved in sucrose apoplast unloading in melon fruit.

### The capacity of fruit sinks to accumulate sugars is independent

**Figure 5:**
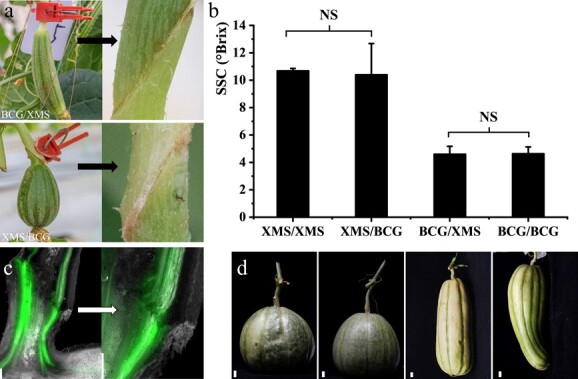
Reciprocal grafting of melon fruits to peduncle proved that the fruit itself determines the fruit sugar content. **a** Reciprocal grafting between low sugar content melon fruits (BCG) and high sugar content melon fruits (XMS). Grafting of BCG fruit to peduncle of XMS (top panels) (BCG/XMS) and grafting of XMS fruit to peduncle of BCG (bottom panels) (XMS/BCG). Fruits from self-grafting are not shown (XMS/XMS and BCG/BCG). **b** Sugar content in reciprocal grafted fruits. NS, no significant difference. **c** CFDA staining shows the vascular cascade status of the grafted wounds at the peduncle. **d** Harvested fruits after grafting, the four photographs (left to right) correspond to the four abscissa columns in the plot shown in panel b. Scale bars = 200 μm in **c** and 1 cm in **d**.

In investigating whether source or sink contributed to the sucrose content differences among melon cultivars, we performed reciprocal grafting of melon fruits and peduncle. The young fruits of the sweet cultivar XMS and the unsweet cultivar BCG were reciprocally grafted to the peduncle to produce mature fruit (35 DAP; Fig. 5a, c, and d). Self-grafting fruit was used as the control. The results showed no significant difference in sugar content of mature fruits whether the fruits were grafted on their own peduncle or another peduncle (Fig. 5b). These results indicate that the fruit sinks are capable of accumulating sugars independently of sources in melon.

### Expression profiling of genes associated with sucrose transport and metabolism during different developmental stages of melon fruit

In determining the mechanism by which the transmembrane sugar transporters and sugar metabolism relative enzymes play a role in the sugar unloading of melon fruit, a wide range of sugar transporter and enzyme genes were determined through melon genomic and RNA-seq databases. A total of 27 related genes (five members from the SWEET family, two members from the SUT/SUC family, two members from the TST family, five members from the HT/STP family, and three members from the NAG family) were found highly expressed in melon fruit.

**Figure 6:**
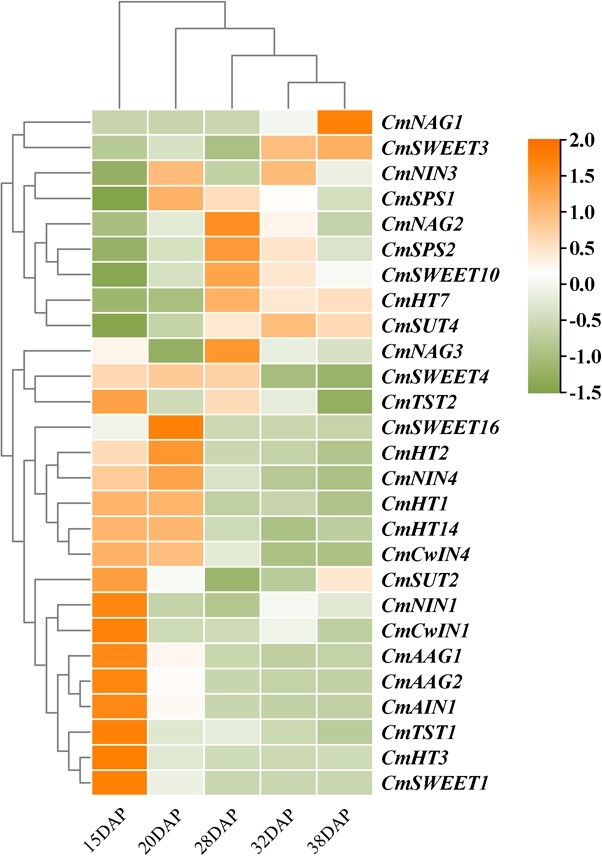
Hierarchical cluster analysis of the expression level of genes involved in sugar transport and metabolism during melon fruit development and maturity. Color scale is shown at the side. Shades of orange indicate higher expression, relative to the mean across samples, whereas shades of green indicate lower expression relative to the mean.

The RNA-seq expression of these genes during fruit development was analyzed to obtain insight into the role of these genes in sucrose accumulation (Fig. 6, Supplementary Data Fig. S5). The results show that the expression level of two SWEET genes (*CmSWEET*3 and *CmSWEET*10), one SUT gene (*CmSUT4*), one HT gene (*CmHT7*), one NAG gene (CmNAG2), and two SPS (Sucrose phosphate synthase) genes (*CmSPS1* and *CmSPS2*) increase in YL melon fruit after 15 DAP when the fruit starts to accumulate sucrose, which indicates that these genes may be involved in sucrose unloading and ripening of melon fruit. In addition, *CmSWEET1/4/16*, *CmSUT2*, *CmTST1*, *CmHT2/3*, *CmAAG1/2*, *CmNIN1/4*, *CmAIN1*, and *CmCwIN1/4* had a relatively high expression at the young stage of melon fruit, and their expression level was downregulated after 15 DAP, which indicated that these genes may be primarily involved in sugar unloading and metabolism during fruit enlargement.

### Expression profiling of genes associated with sucrose transport and metabolism enzyme genes in melon cultivars with different sugar content in fruits

**Figure 7:**
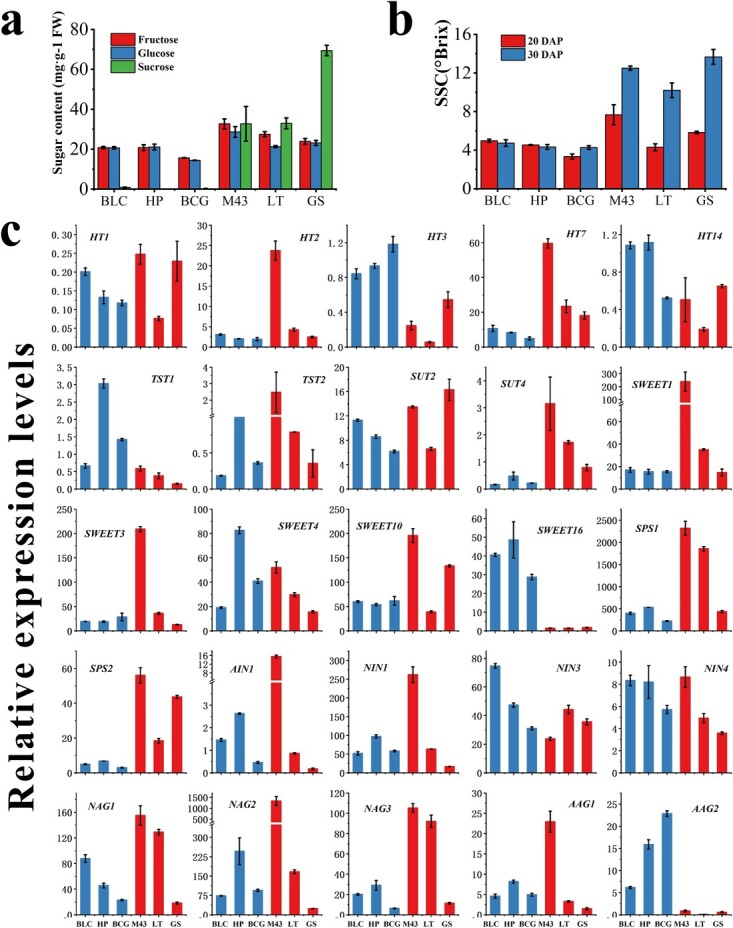
Expression analysis of genes related to sugar transport and metabolism in different genotypes of melon fruits. **a** SSC in melon fruits of different genotypes. **b** Glucose, fructose, and sucrose contents in fruits of different melon genotypes. **c** qRT–PCR analysis of relative expression of 11 sucrose metabolism-related genes (*AAG*s, *NAG*s, *AIN1*, *NIN*s, and *SPS*s) and 14 sucrose transport genes (*SWEET*s, *HT*s, and *SUT*s) in melon fruits. TUA (α-tubulin) was used as the reference gene. Error bars represent ± standard error for three technical replicates of three biological replicates.

Melon has a rich variety of resources. Although most cultivated varieties are sweet, there are still some unsweet melon fruits that are used for cooking. In identifying the differences in sugar accumulation patterns between sweet melon and unsweet melon varieties, we compared the differences in sugar content and the expression of genes related to sugar metabolism and transport in the fruits of three sweet and three unsweet varieties. The soluble solids content (SSC) of fruits of sweet melon and unsweet melon showed no significant difference at 20 DAP. However, the SSC in sweet varieties (M43, LT, and GS) was significantly higher than that in unsweet varieties (BLC, HP, and BCG) at 30 DAP (Supplementary Data Fig. S6). Further analysis of sugar components of melon fruits (30 DAP) showed that sucrose was the major component of sugar in sweet varieties, whereas sucorse content of unsweet varieties was almost zero (Fig. 7b), which indicates that sucrose content is a key factor in determining sweet and unsweet varieties. In determining the genes that may be responsible for this difference, we quantitatively analyzed the expression of genes related to sugar unloading and transport in fruits of these varieties. The results showed that the expression of four sugar transporter genes (*HT7*, *SUT4*, *SWEET1*, and *SWEET10*) and five sugar decomposition or synthetase genes (*SPS1*, *SPS2*, *NAG1*, *NAG2*, and *NAG3*) in sweet varieties was higher than that in unsweet varieties (Fig. 7c). Moreover, three sugar transporter genes (*HT3*, *TST1*, and *SWEET16*) and one sugar decomposition gene (*AAG2*) were highly expressed in unsweet varieties compared with sweet varieties (Fig. 7c).

**Figure 8:**
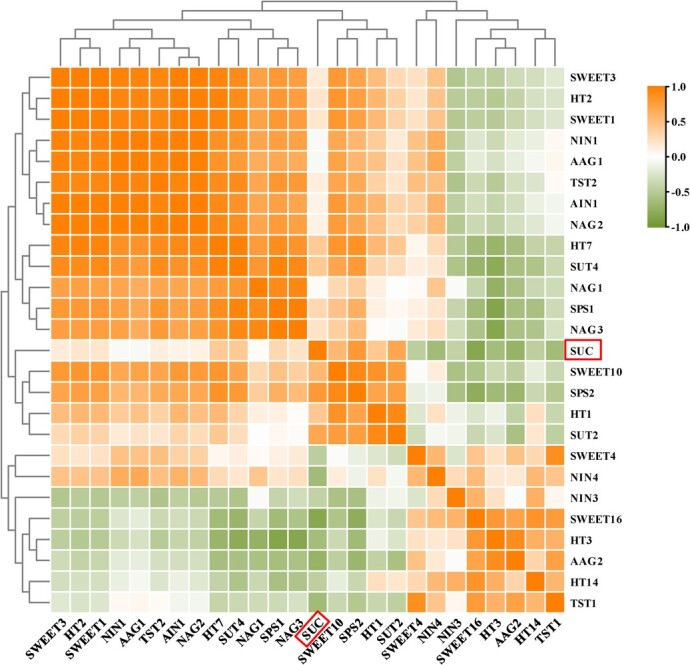
Correlation analysis of sucrose content and gene expression level of genes related to sugar transport and metabolism in 30 DAP melon fruit. The correlation matrix is calculated by using data on sugar content and gene expression among varieties (BCG, BLC, HP, M43, GS, LT) through SPSS. Color scale is denoted at the side. Shades of orange indicate higher expression, relative to the mean across samples, whereas shades of green indicate lower expression relative to the mean.

Furthermore, based on the sucrose content and expression level, correlation analyses were performed to reveal the relationship between sucrose content variation and gene expression in fruits. The constructed correlation heat map showed that *CmSUT2*, *CmSUT4*, *CmHT7*, *CmHT2*, *CmSWEET10*, and *CmSPS2* were highly and positively correlated with sucrose content, whereas *CmSWEET16*, *CmTST1*, *CmNIN4*, *CmAAG2*, and *CmHT3* were negatively correlated with sucrose content among melon cultivars (Fig. 8).

### CmSWEET10 is a key sucrose transporter involved in the phloem apoplast unloading of sweet melon fruit

**Figure 9:**
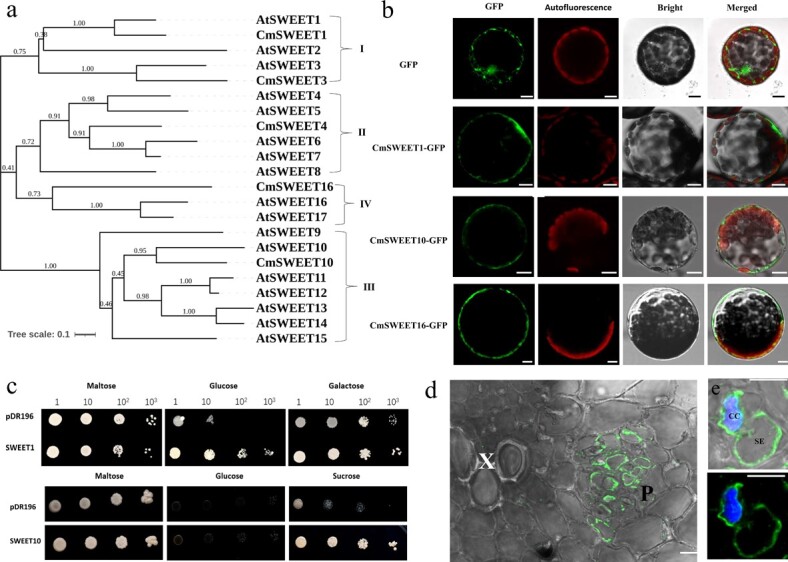
Functional identification of SWEET proteins in melon fruit. **a** Phylogenetic tree of melon (Cm) and *Arabidopsis* (At) SWEET proteins. **b** Subcellular localization of CmSWEET1/10/16-GFP in tobacco protoplasts. **c** Growth recovery on glucose and galactose of EBY.VW4000 (hexose transporter-deficient yeast cells) by *CmSWEET1* expression, and growth recovery on glucose and sucrose of CSY4000 (hexose/sucrose transporter-deficient yeast cells) by CmSWEET10 expression. **d** Immunofluorescence localization of CmSWEET10 in transverse sections of melon fruit. Green staining shows CmSWEET10 localization. **e** CmSWEET10 plus histochemical staining of a nucleus with DAPI (blue) in a cross-section of melon fruit SE–CC. X, xylem; P, phloem; SE, sieve element; CC, companion cell; GFP, green fluorescence protein; Bars = 10 μm.

Sucrose transporters, such as SWEET and SUT, are involved in sucrose apoplasmic unloading and accumulation in melon fruits (Figs 4, 6, and 7). In determining which SWEET gene plays a key role in this process, five SWEET protein members expressed in melon fruit were analyzed. An unrooted phylogenetic tree of 5 CmSWEET and 17 AtSWEET protein sequences was constructed by using MEGA X with a bootstrap method (Fig. 9a).

These SWEET protein sequences were clustered into four clades based on the classification by Chen *et al*. [34] In detail, clade I contains CmSWEET1, CmSWEET3, and three AtSWEETs (AtSWEET1–3). Clade II contains CmSWEET4 and five AtSWEETs (AtSWEET4–8). Clade III contains CmSWEET10 and seven AtSWEETs (AtSWEET9–15). Clade IV contains CmSWEET16, AtSWEET16, and AtSWEET17.

The subcellular localization results showed that CmSWEET1 and CmSWEET10 were localized to the plasma membrane, and CmSWEET16 was localized to both plasma membrane and tonoplast (Fig. 9b, Supplementary Data Fig. S7). In investigating the transport properties of CmSWEET proteins, the hexose uptake-deficient yeast mutant *EBY.VW4000* and the hexose/sucrose transporter-deficient yeast mutant *CSY4000* were used. The open reading frame (ORF) sequence of *CmSWEET1* or *CmSWEET10* was subcloned into the yeast expression vector pDR196. A drop test showed that yeast with CmSWEET1 expression can grow well on 2% (w/v) glucose and galactose (Fig. 9c), thereby demonstrating that CmSWEET1 is a glucose and galactose transporter. Meanwhile, in the CSY4000 mutant, the clade III SWEET protein CsSWEET10 conferred sucrose uptake but did not allow hexose uptake.

Given the high expression of CmSWEET10 in sweet melon fruit and its sucrose uptake activities, we performed immunohistochemical analysis to determine its cell-type localization in melon fruit tissues. Using an Alexa Fluor^®^ 488-labeled secondary antibody, CmSWEET10 was detected exclusively in the SE–CC (Fig. 9d and e). Pre-immune serum-probed sections showed no labeling (Supplementary Data Fig. S8). This finding was consistent with the result from immunohistochemical staining using alkaline phosphatase (Supplementary Data Fig. S9).

## Discussion

### Phloem unloading pattern of melon fruit changed during cultivation and domestication

Sucrose produced by photosynthesis in higher plants is transported to sink organs through the phloem, and the results of transport and distribution directly affect the growth, yield, and quality of plants [2]. In fruits with high concentration of soluble sugar, most of them unload photoassimilation products from the vascular bundle through the apoplasmic pathway, such as pear [3], apples [4], and grapes [5]. This process ensures efficient phloem transport, and it is crucial for sugar accumulation in the fruit [6, 35]. In particular, Cucurbitaceae plantsare typically plants in which RFO transport occurs. The main transport forms of photosynthate in the phloem are raffinose and stachyose [30, 36]. Based on reported studies of Cucurbitaceae, the phloem unloading pathway in cucumber and watermelon fruit of cultivated species was apoplasmic [25, 30]. In our study, CFDA staining and plasmodesmata density counting indicated that the phloem unloading pathway during cultivated melon fruit developing switches from symplasmic to apoplasmic (Figs 1 and 3, Supplementary Data Fig. S3). The phloem unloading pathway in wild melon fruit is mainly symplastic during fruit development (0–30 DAP).

Compared with wild melons, the fruits of most cultivated melons are significantly larger and sweeter (Supplementary Data Fig. S1). The cultivated melon fruit has a rapid enlargement stage and a rapid sucrose accumulation period during melon fruit development. The phloem apoplast unloading pathway is conducive to the reverse concentration gradient transport of assimilates, thereby promoting the accumulation of high-concentration sugars [33]. Therefore, the change in phloem unloading pattern of cultivated melon fruit may occur during long-term cultivation and selection. Zhao *et al*. [32] found that many key sugar transporters (HT7, SWEET1, SUT2/4) involved in the fruit phloem apoplasmic unloading pathway have undergone domestication selection. This may be related to the shift in the unloading pathway of cultivated melon.

### Identification of key genes involved in phloem assimilate apoplast unloading in melon fruit

The yield and sugar content of melon are determined by two important fruit developmental stages: the expanding and premature stages. At the expanding stage, the mesocarp, endocarp, and placenta diameters increase rapidly, resulting in an enlargement of the fruit. After that, the sucrose content increased rapidly at the premature stage [11]. The accumulation of sucrose depends on upregulation of SPS expression in combination with the decreased expression level of invertase (acid invertase and cell wall invertase, AIN and CwIN) [22–24]. Notably, these enzymes on the unloading path are localized on different regions of different cells, with NAG often localized to the companion cell [31]. NIN (neutral invertase)  and SPS are localized in the cytoplasm, AIN to the vacuole, and CwIN to the cell wall. The bulking and sucrose accumulation of melon fruits are dependent on efficient sugar unloading, which indicates not only the shift of the unloading pathway but also the reliance on the function of sugar transporters.

As an active transport process, the apoplasmic pathway requires sugar transporters to transport sugar to the apoplasmic space and then into parenchyma cells [37]. This is also supported by esculin staining and the expression of the sugar transporters in developing melon fruits (Figs 4 and 6). In the apoplasmic pathway, the transmembrane transport of sucrose is catalyzed by various sugar transporters [38]. These transporters, together with metabolic enzymes, regulate fruit development and ripening. In recent years, studies on the expression of *HT*s in organs have increased, showing accumulation of high concentrations of hexose, such as in sugar beet taproots [12] and fruit flesh, e.g. apple [13], strawberry [14], peach [15], and apricot [16]. In apple fruit, sucrose enters the PCs via the apoplasmic pathway after being released from the SE–CC and contributes to sucrose import via hydrolysis into hexose by CwIN [4, 40]. High HT activity is required in apple fruit to transport hexose from the cell wall to PCs against the hexose concentration gradient [40, 41]. However, little is known about HT in the fruit of Cucurbitaceae plants. We identified five *HT*s and two *CwIN*s expressed in melon fruit (Fig. 6, Supplementary Data Fig. S5). *CmHT2/3/7* and *CmCwIN1/4* were primarily expressed at the expanding stage, and only *CmHT7* was expressed at the premature stage. Studies have demonstrated that these hexoses produced in the expanding stage are usually involved in developmental processes such as cell division [42]. *SUT2* is expressed in dicots, and it is important for the apoplasmic unloading pathway of sucrose [43]. SUT4 is thought to be localized to the vacuolar membrane, and its expression is negatively correlated with the cytosolic sucrose concentration, which is important for cellular sugar storage and maintenance of cytoplasmic osmolarity balance [44]. SWEET is a newly identified class of sugar transporters with pH-independent transport capabilities [45]. Among the five *SWEET* genes identified from melon fruit, *CmSWEET1/4/16* were the primarily expressed SWEETs at the expanding stage, whereas *CmSWEET3/10* were expressed at the premature stage (Fig. 6, Supplementary Data Fig. S5). It does not seem that SWEET protein membership in a clade predicts its physiological function [7]. Thus, we performed functional analysis of SWEETs to comprehensively understand the function of sweet proteins in unloading melon assimilates. Similar to *AtSWEET1*, *CmSWEET1* exhibited glucose and galactose transport activity, and it was localized at the plasma membrane (Fig. 9b and c) [45]. SWEET10, which belongs to clade III, may play an important role in the accumulation of sugar in grape [46] and citrus fruits [47]. As the main *SWEET* expressed in the premature stage of melon fruit development, CmSWEET10 exhibited sucrose transport activity, and it was localized at the plasma membrane (Fig. 9b). Further immunolocalization results showed that CmSWEET10 was expressed in the CC-SE of melon fruit, which indicates that it may play a crucial role in sucrose unloading in melon fruit phloem (Fig. 9d and e).

The function of the unloading genes expressed at the fruit expanding stage is primarily to metabolize assimilated products to hexoses, which is beneficial for fruit biosynthesis, thereby leading to rapid fruit expansion. During the premature stage, the expression of invertase decreases and that of SPS and specific sucrose transporters increases, indicating that assimilate unloading is stored in the vacuole in the form of sucrose.

### Differential expression of sugar transporter in melon led to the difference in sugar content among cultivars

In thousands of years during the cultivation history of melon, melon underwent three independent domestication events, which resulted in the diverse characteristics of the cultivars [32]. These diverse characteristics are reflected not only in the rind sutures and peel and flesh color, but also in the sugar content, which is the most important trait. Sucrose content primarily causes the difference in SSC among melon cultivars (Fig. 7b). The discrepancy in sugar content was previously thought to be due to the differences in sucrose metabolism in the premature stage of fruit development. Our study confirmed this notion. Recently, the key sucrose metabolism enzymes have been shown to be also regulated differentially by sugar transporters [13, 28]. The alteration of key genes during phloem unloading may also contribute to the differential sugar content of melon fruits from cultivars during domestication, which was supported by recent findings in grape [5], tomato [48], and watermelon [29–31]. Ren *et al*. [29–31] demonstrated that several key phloem unloading genes (*ClAGA2*, *ClSWEET3*, *ClTST2*, and *ClVST1*) have been selected during the domestication of watermelon, which has led to the modern sweet watermelon evolving from a non-sweet ancestor during domestication. Some of these genes related to phloem unloading (*AAG2*, *NAG2*, *HT7*, *SPS1/2*, *SWEET1*, *SUT2/4*, *TST2*) were found to be within the putative domestication sweeps in cultivated melons [32]. The differences in sugar content among melon cultivars may also be due to the different expression of genes related to phloem unloading. We determined the expression of the identified transporters and sucrose metabolism in fruits from six cultivars with different sugar contents (Fig. 7). The genes related to phloem unloading in the low-sugar cultivars were consistently expressed and significantly different from those in sweet cultivars. Differences in the expression of phloem unloading genes exist among sweet cultivars, indicating that different genes are selected during melon domestication in different regions, which was confirmed in a previous population genetics study [32]. Furthermore, we correlated sucrose content with the expression of these genes and showed that *SUT2*, *SUT4*, *HT7*, *HT2*, *SWEET10*, and *SPS2* were positively correlated with sucrose content, whereas *SWEET16*, *TST1*, *NIN4*, *AAG2*, and *HT3* were negatively correlated with sucrose content (Fig. 8). The transcript level of *CmTST2* is not significantly and positively correlated with sucrose content, indicating that *CmTST2* may be regulated post-transcriptionally. In cotton, the function of GhTST2 is regulated by its interaction with CBL2 and CIPK6 [49]. A preliminary result illustrates interspecific expression differences in phloem unloading genes. Functional characterization of more relevant genes and identification of transcription factors associated with phloem transport are necessary, which may allow the establishment of a deeper relationship between these genes and sucrose content.

### Model of phloem unloading at the expanding and premature stages

Combining previous research and our recent findings, we propose a model (Supplementary Data Fig. S10) of phloem unloading in the expanding stage versus the premature stage in sweet melons. High anabolism and catabolism of sugar are observed at the beginning of fruit development, which are the metabolic processes required for carbon skeleton construction and energy supply in plants [50]. During the expanding stage of melon, NAG2, NAG3, AAG1, and AAG2 initially hydrolyze RFOs and then NIN1 and NIN4 hydrolyze sucrose into hexoses. These hydrolysate products of RFOs can be expelled into the apoplast space through SWEET4 and SWEET16, where sucrose is partly decomposed by CwIN1 and CwIN4. Then sucrose and hexoses are imported into the PP cells in concert with SUT2, HT2, HT3, and HT7. Finally, the sucrose in the PP cells is transported into the vacuole by TST1 or TST2 and broken down into hexoses by AIN1. The products of phloem unloading during the expanding stage are dominated by monosaccharides and primarily used for the rapid growth and development of cells, thereby promoting rapid expansion during the expanding stage of melon.

At premature stages of fruit development, RFOs are primarily hydrolyzed by NAG2. Meanwhile, the transcription of AAGs and NINs is downregulated. The hydrolysates are rarely hydrolyzed, but are directly transported into the parenchyma cells through the apoplastic route. Sucrose is transported through SE–CC to PP cells by SWEET10 and SUT2. Hexose is initially transported to PP cells by SWEET3 and HT7 and then irreversibly synthesized into sucrose by SPS1 and SPS2. Finally, TST2 and SUT4 simultaneously promote vacuolar sucrose accumulation and maintain osmolarity balance. In this stage, sucrose is primarily unloaded in the phloem, and most of the assimilated products become sucrose, which eventually leads to a rapid accumulation of the sugar content of melon.

## Materials and methods

### Plant materials

Melon [*C. melo* L., cultivated varieties: cv. Balengcui (BLC), cv. Huapicai (HP), cv. Baicaigua (BCG), cv. M1–43 (M43), cv. Longtian-4 (LT), cv. Gaoshicui (GS), cv. Elizabeth (YL), cv. Xiaomaisu (XMS), cv. Yangjiaosu (YJS), cv. Yuniang (YN); wild varieties: Jinkouye (JKY), Mapaogua (MPG)] (Supplementary Data Fig. S1). During the growing season of March to June or July to November, plants were grown in polytunnels. Melon fruits at different developmental stages between 0 and 40 DAP were harvested for RNA-seq, RT–qPCR and sugar quantification. All samples were immediately frozen in liquid nitrogen and stored at −80°C.

### Loading of carboxyfluorescein diacetate, esculin, and Texas red

A solution of non-fluorescent, membrane-permeant dye CFDA (Sigma–Aldrich) was introduced into melon fruits in accordance with the method of Zhang *et al*. [4]. The fruit-bearing detached shoots were cut from the plant and immediately recut under EDTA solution (50 mM) to prevent wound healing. The wounded shoots were immersed in a tube with 1 ml of 1 mg/ml aqueous solution and illuminated in a growth chamber at 25–27°C for 24 hours. Afterwards, the fruit tissues were sectioned and examined under a confocal laser scanning microscope (CLSM, Olympus FV1000 or Leica SP8). Sampling sites are shown in Fig. 3a and b.

In distinguishing the xylem from the phloem under a CLSM, tissues from CFDA-treated fruits were treated with Texas red dextran (Invitrogen) as described by Hu *et al*. [25]. CFDA and Texas red dextran were observed at 488 and 543 nm excitation wavelengths, respectively, under a CLSM.

In showing the role of sugar transporters in assimilate unloading of fruit, plants were allowed to translocate the CF for 24 hours and esculin for 1 hour. Afterwards, the fruit tissues were sectioned and examined under a confocal laser scanning microscope.

### Tissue preparation for ultrastructural observation and measurement of plasmodesmal density

Young melon fruits (0 DAP) and premature melon fruits (20 DAP) were cut into small pieces of ~0.2 mm × 0.2 mm size and prepared for ultrastructural observation, which was conducted as previously described by Xie *et al*. [51]. At least three observations were made for each ultrathin section. We counted plasmodesmata at all cell interfaces, including those between CC and SE, PP and SE, and PP and PP. Counting plasmodesmata was performed on a transverse section of specific interface lengths of cell/cell interfaces to obtain the plasmodesmal density (number of plasmodesmata µm ^−1^).

### Fruit grafting

The sweet cultivar XMS and unsweet cultivar BCG were used to perform reciprocal grafting. Young fruits (4–5 DAP) of XMS were grafted onto the peduncle of BCG after bevel cutting the fruit stalk and vice versa (Fig. 4a). Grafted fruits were bagged with an aluminum foil bag to maintain moisture and protect from light for 7 days and fixed with a clamp until mature. In total, 100 ovaries were grafted, with a survival ratio of ~25%. At 30 days after grafting, grafted fruits were harvested for SSC measurement.

### Determination of sugars

The SSC of melon fruits was measured using a pocket digital refractometer (Pocket PAL-1, Atago) and the data are listed in Supplementary Data Table S2. Contents of different sugars (fructose, glucose, and sucrose) were determined and analyzed by high-performance liquid chromatography (UltiMate 3000) using an evaporative light-scattering detector (Alltech ELSD6000as) as described by Vizzotto *et al*. [52]. In brief, mature fruit tissues of different varieties were sampled and stored at −80°C before use. In addition, 1–2 g of pulverized sample was ultrasonically extracted in 80% (v/v) ice-cold methanol at 70°C for further detection under the following conditions: column, Welch Ultimate HILIC Amide (4.6 × 250 mm, 5 μm); mobile phase, 0.02% ammonia–acetonitrile (25:75) isometric elution; flow rate, 1.0 ml/min; column temperature, 40°C. Data on each sugar were collected. For the standard curve, pure sugars were quantified using methyl-α-d-glucopyranoside as internal standard. They were also used after the dilution reached 1, 2, 4, 6, and 8 mg/ml. Then, we calculated the sugar content using Chromeleon 7.0.

### Identification and screening of genes involved in sugar metabolism and transport

In determining the putative sugar transporters (SWEET, HT, and SUT) and sugar metabolic enzymes (AAG, NAG, AIN, NIN, and SPS) that are responsible for sugar unloading and accumulation in melon, we BLAST-searched the translated melon genome (http://cucurbitgenomics.org/) with the homologous sequences of *Arabidopsis thaliana*. The genes that have relatively high expression in melon fruits based on the RNA-seq data from the Cucurbit Expression Atlas (projects PRJNA286120, PRJNA288543, and PRJNA383830) were selected as candidates. The line chart (Supplementary Data Fig. S5) and heat map (Fig. 6) of candidate genes in Elizabeth melon during several developmental stages were derived from our previously published data [53]. The candidate gene information is listed in Supplementary Data Table S3.

### RNA extraction and RT–qPCR

According to the manufacturer’s instructions, total RNA was extracted from specific tissues using TRIzol (Transgen). SuperScript III reverse transcriptase (Invitrogen) and RNase H (Invitrogen) were used to reverse-transcribe the RNA. The cDNA was used as template for RT–qPCR analysis. The gene-specific qPCR primers used are listed in Supplementary Data Table S4. RT–qPCRs were performed with a 10-μl reaction on an ABI7500 system (Bio-Rad). The expression level of genes was analyzed using the 2^−ΔΔ^Ct method [54].

### Construction of the phylogenetic tree of SWEET genes in melon

A phylogenetic dendrogram was constructed using MEGA X [55] based on bootstrapping with 1000 replications, adopting the Poisson correction distance. The sequences are listed in Supplementary Data Table S3 with their GenBank accession numbers and sources.

### Heterologous expression of *CmSWEET1*/*10* in yeast for functional analysis

The ORF of *CmSWEET1/10* was cloned into pDR196, a yeast expression vector, to test the functionality of *CmSWEET1/10* [56]. Construction of vector was performed as described [57]. PCR products were digested with either SmaI or XhoI to yield *CmSWEET1/10*. The fragment was subcloned into the corresponding restriction sites of pDR196 to yield pDR196/*CmSWEET1/10*, and the construct was confirmed by sequencing. EBY.VW4000 (hexose transporter-deficient yeast strain) was transformed with pDR196/*CmSWEET1*, and CSY4000 (sucrose and hexose transporter-deficient yeast strain) was transformed with pDR196/*CmSWEET10* using the method of Morita and Takegawa [58]. pDR196 was used as a control. The drop test for yeast growth was performed as previously described [59].

### Subcellular localization of CmSWEET proteins

CmSWEET1/10/16-GFP fusion vectors were constructed by using the vector pH7LIC5.0- ccdB rc- N- eGFP, and the specific primers are provided in Supplementary Data Table S1. The subcellular localization of CmSWEET1, CmSWEET10, and CmSWEET16-GFP was performed by transient expression in *Nicotiana benthamiana* epidermis as described [57]. Expression of *CmSWEET16*-GFP in melon fruits was performed by transient expression and was performed according to Cheng *et al*. [57]. As a control, the empty vector expressing untargeted GFP was used. The fluorescence was imaged using CFLM (confocal laser scanning microscopy; Olympus FV1000 or Leica SP8).

### Immunolocalization of CmSWEET10

Immunolocalizations of melon fruits and DAPI staining were performed as described [60]. Antibody was detected by IgG-Alexa Fluor^®^ 488 (green fluorescence; Abcam). Antibody-decorated sections were imaged using CLSM (Leica SP8).

### Statistical analysis

All data were expressed as means ± standard errors of triplicate experiments. Transcript levels [FKPM (fragments per kilobase of transcript per million mapped reads)] were analyzed from the database of Cucurbit Genomics (https://cucurbitgenomics.org/). The correlation matrix was calculated through SPSS. Figures were constructed via Origin Pro 2018 and TBtools [61].

## Acknowledgements

We thank Professor Huaisong Wang (Chinese Academy of Agricultural Sciences) for providing melon seeds and thank Professor Chunlong Li (Huazhong Agricultural University) for providing yeast strain CSY4000. We thank Muhammad Mohsin Kaleem for his critical reading and editing of the manuscript. We also thank Jianbo Cao and Limin He (Huazhong Agricultural University) for transmission electron microscope support. This work was supported by the National Natural Science Foundation of China (31972435), the National Key Research and Development Program of China (2019YFD1000300), the Fundamental Research Funds for the Central Universities (2662018QD062), and the China Agriculture Research System of MOF and MORA (CARS-25).

## Author contributions

J.C., Y.Z., and Z.B. conceived and designed the experiments. Y.Z. performed most of the experiments and analyzed the data. K.L. identified the functional characteristics and subcellular localization of SWEET protein. D.Y., P.Z., and J.C. performed the CFDA and tracing experiment. S.W. measured the sugar content. Z.W. and Y.Z. performed the qRT–PCR. Y.Z. and J.G. conducted the fruit grafting experiment. Y.Z. prepared the figures and tables and wrote the first draft of the manuscript with assistance from S.W. J.C. revised the manuscript and figures.

## Data availability

Relevant data can be found within the paper and its supporting materials. All data from this study are available from the corresponding author upon reasonable request.

## Conflict of interest

The authors declare that they have no known competing financial interests or personal relationships that could have appeared to influence the work reported in this paper.

## Supplementary data

Supplementary data are available at *Horticulture Research* online.

## Supplementary Material

Web_Material_uhad123
