# Supplementary material for: Phloem unloading in cultivated melon fruits follows an apoplasmic pathway during enlargement and ripening
Source: Hortic Res. 2023 Jul 4;10(8):uhad123. doi: 10.1093/hr/uhad123 (PMC10405131; doi:10.1093/hr/uhad123)
Supplement: Web_Material_uhad123 [file web_material_uhad123.zip › Supplemental Figures.docx]

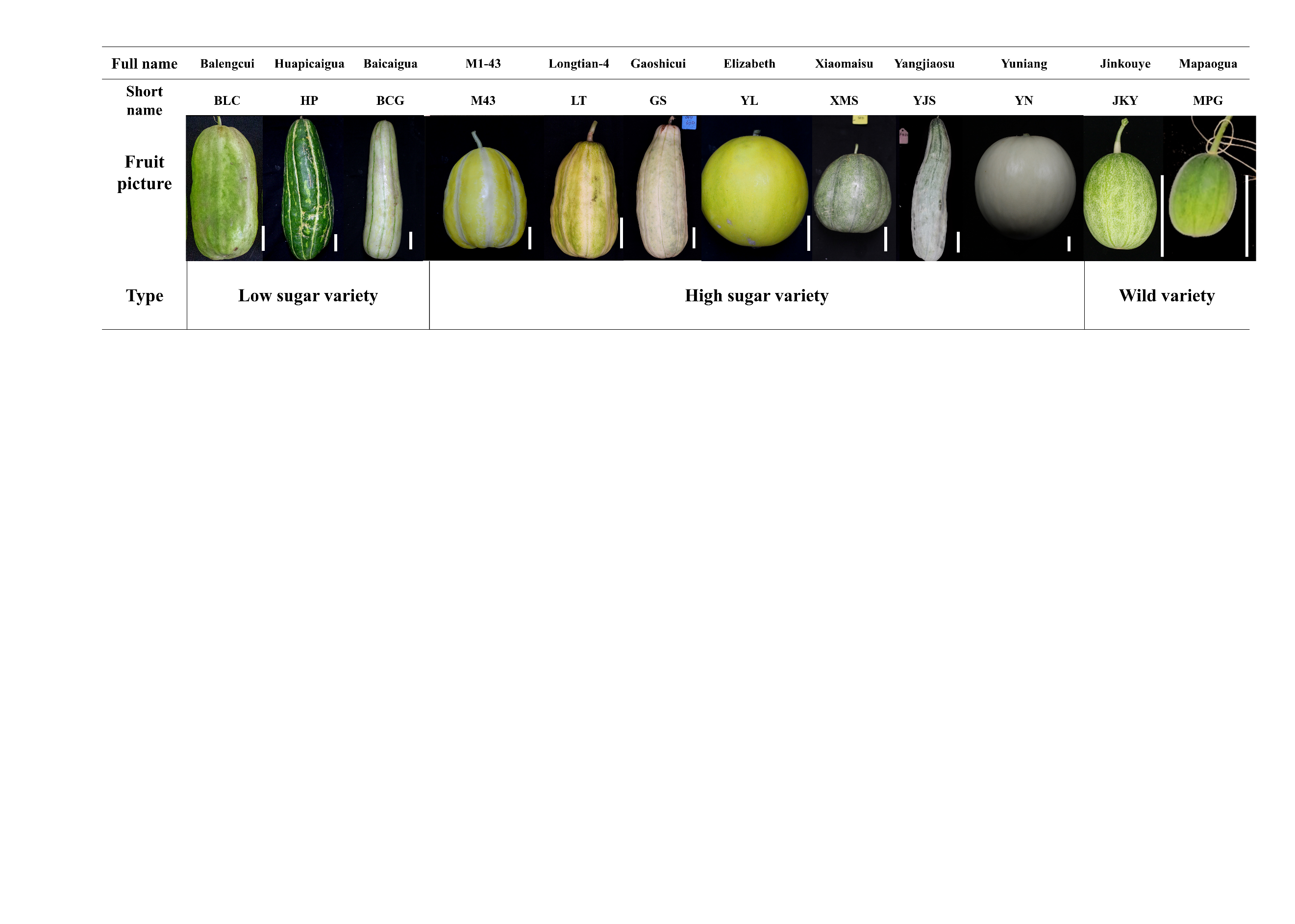


**Fig. S1 Fruits of melon varieties used in this study.** From left to right there are cv. Balengcui (BLC), cv. Huapicai (HP), cv. Baicaigua (BCG), cv. M1-43 (M43), cv. Longtian-4 (LT), cv. Gaoshicui (GS), cv. Elizabeth (YL), cv. Xiaomaisu (XMS), cv. Yangjiaosu (YJS), cv. Yuniang (YN), cv. Jinkouye (JKY) and cv. Mapaogua (MPG). Bars = 5 cm


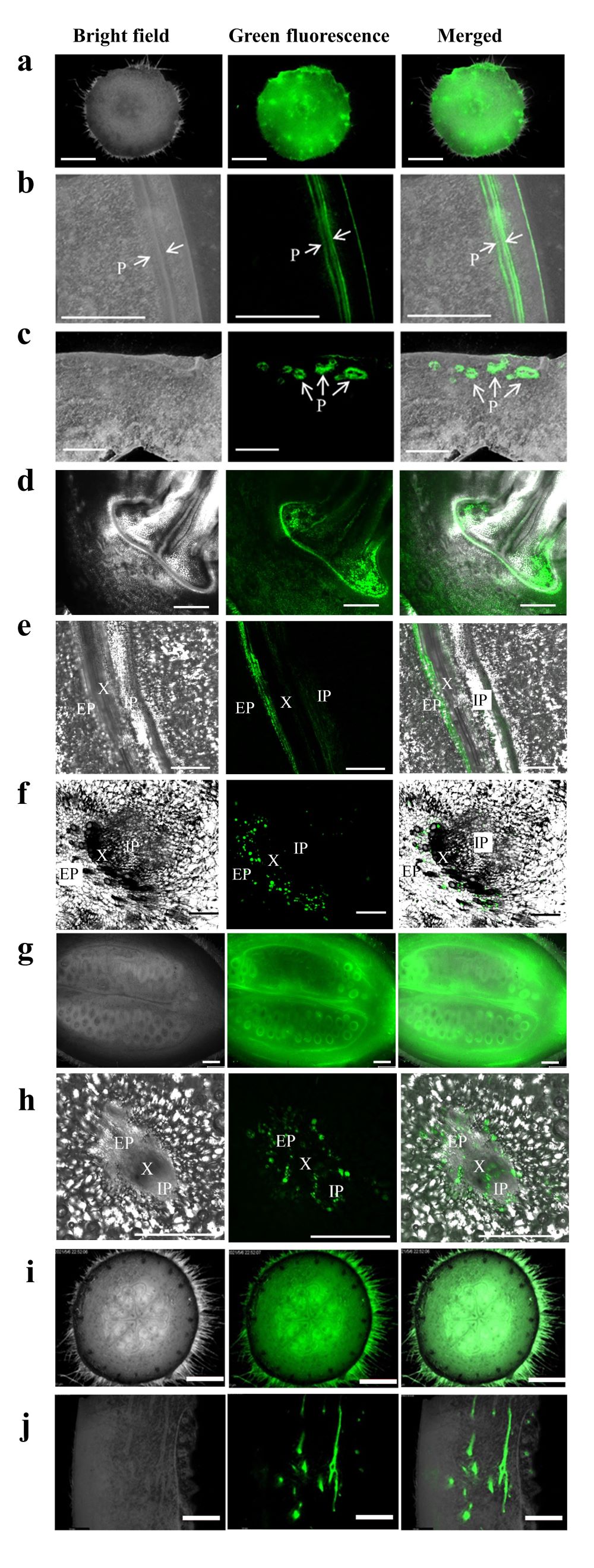


**Fig. S2 CF transport imaging in melon fruits.** a-c, m1-43; d-f, YJS; g-h, XMS, i-j YN. a, d g and i, represent the fruits that younger than 5 DAP (0-5 DAP); b, c, e-f, h and j, represent the fruits that older than 10 DAP (10-30 DAP); a, c, d, f, h-i, transection; b, e, g, and j, longitudinal section. DAP, days after pollination; EP, external phloem; IP, internal phloem; P, phloem; X, xylem. Bar in a, g, i, and j = 10 mm; Bars in b-d = 2 mm**;** Bars in e-f, h = 200 µm.


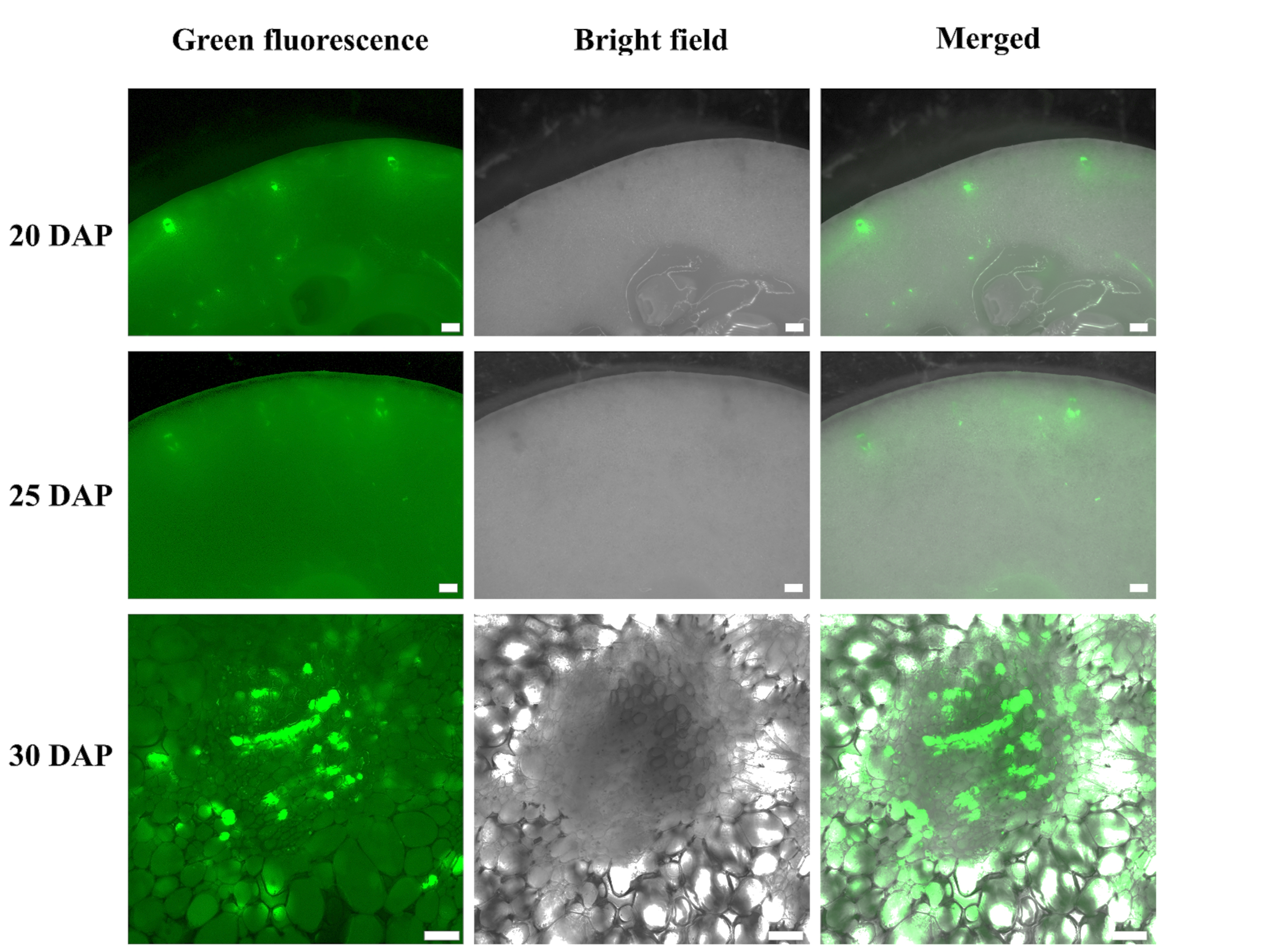


**Fig. S3 CF transport imaging of JKY during fruit development.** Bars in 20 and 25 DAP=1 mm, in 30 DAP=50 μm. DAP, days after pollination.


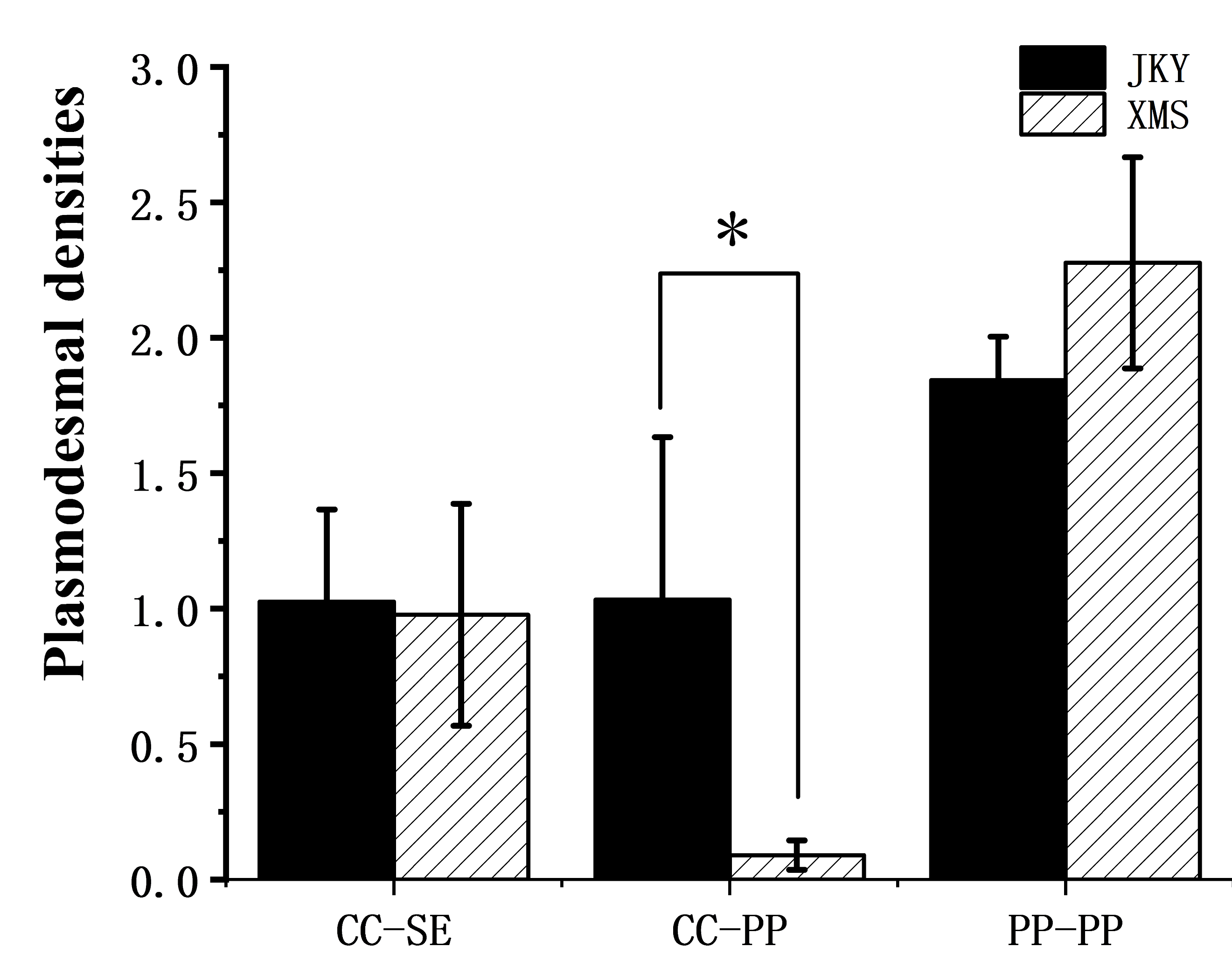


**Fig. S4** **Difference of plasmodesmata density in the fruits (20 DAP) of XMS and JKY.** Each value represents the mean ± SD of three replications. * *t* test significant at *P* < 0.05


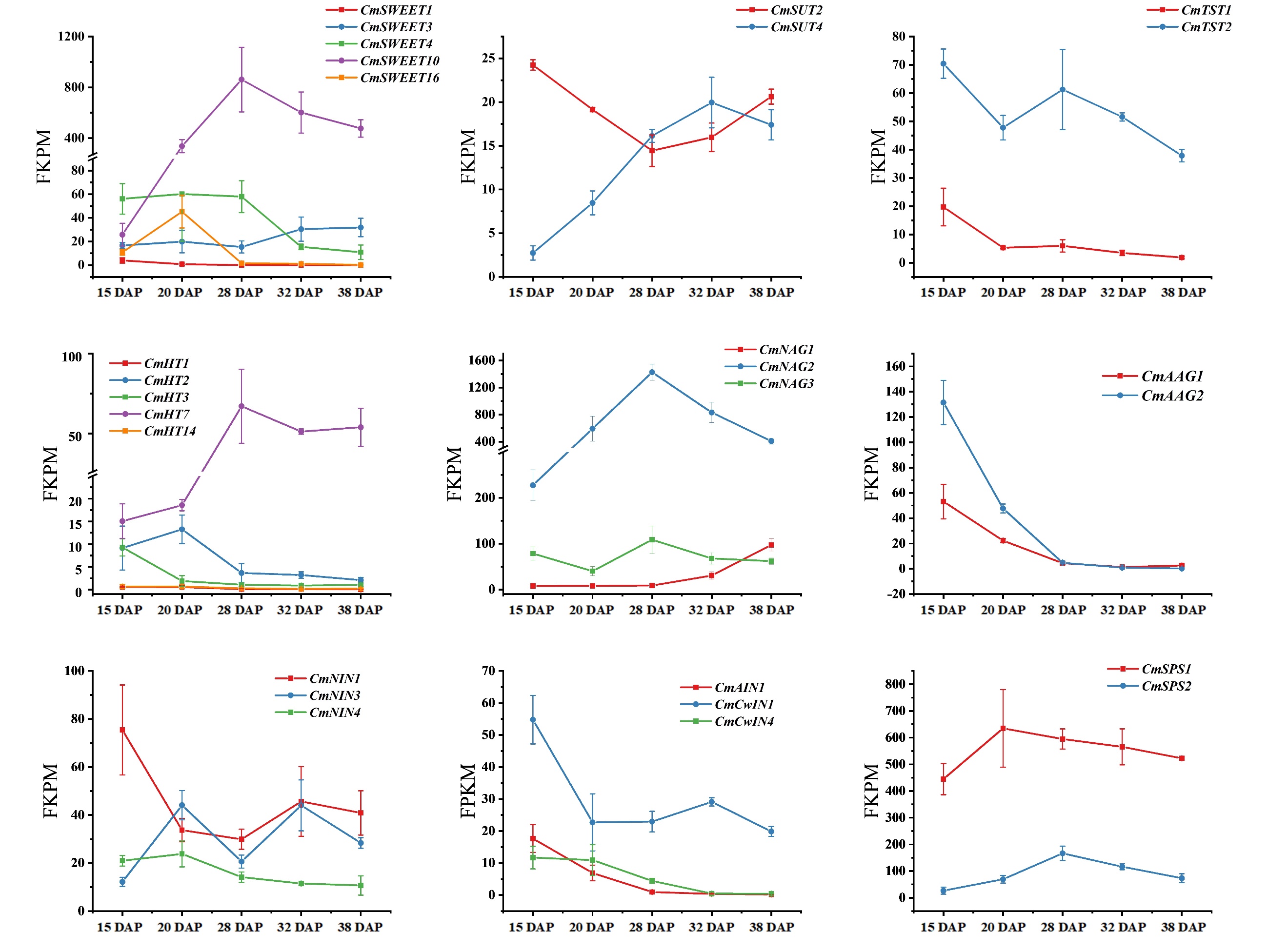


**Fig. S5** **Expression analysis of genes related to sugar transport and metabolism in Elizabeth melon fruits during different developmental stages.** DAP, days after pollination. FPKM: Fragments Per Kilobase of exon model per Million mapped fragments. Mean values ± SE of three independent biological replicates are given.


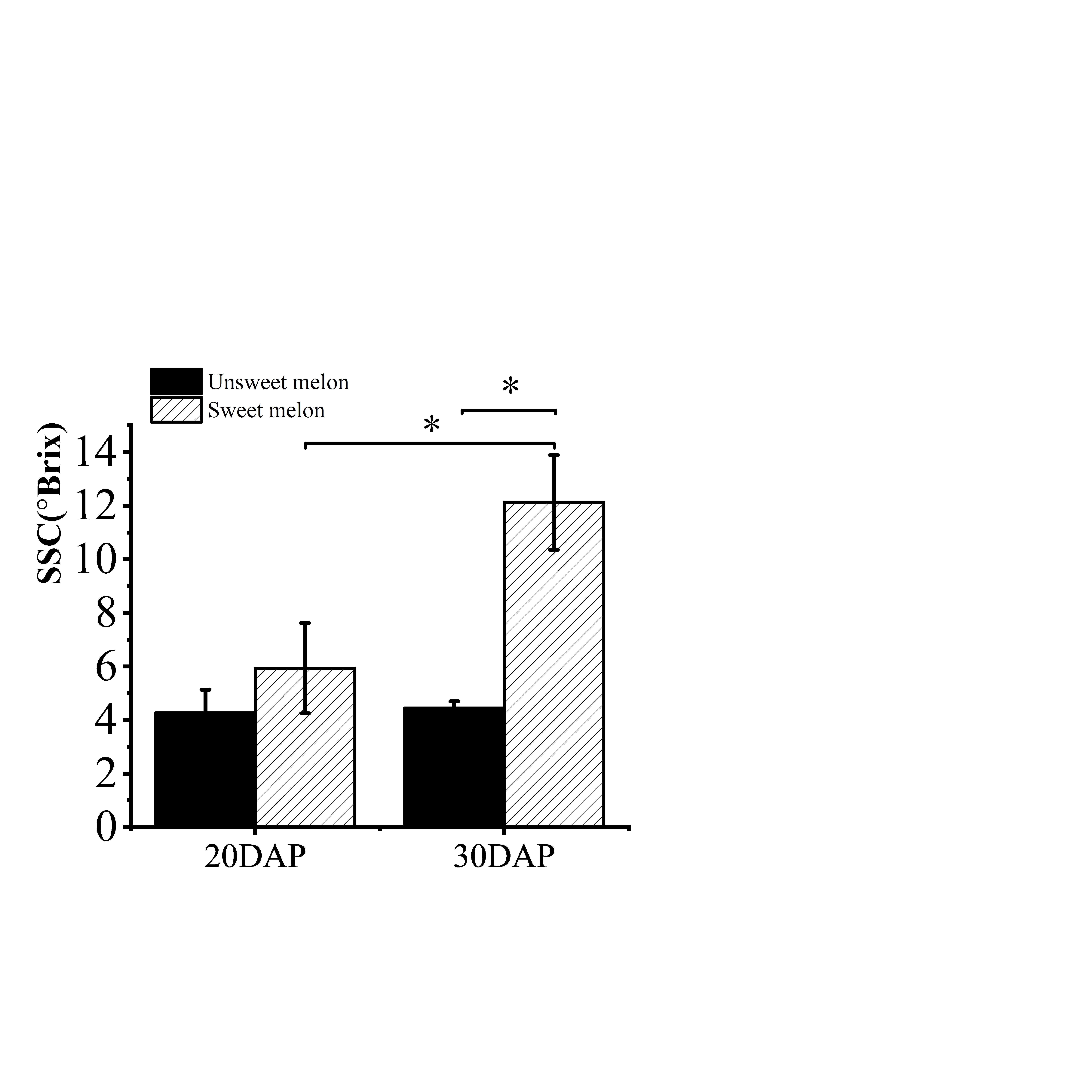


**Fig. S6 SSC** **difference between sweet melon (M43, LT, GS) and un-sweet melon (BLC, BCG, HP) at 20 and 30 DAP.** Each value represents the mean ± SD of three varieties. * *t* test significant at *P* < 0.05. DAP, days after pollination.


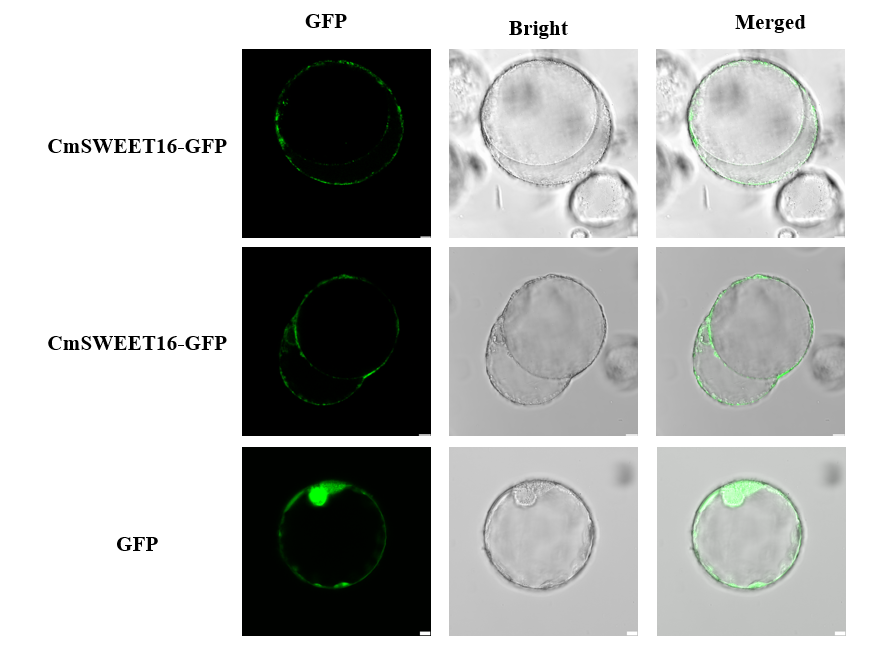


**Fig. S7** **Localization of CmSWEET16-GFP in melon fruit protoplast.** The CLSM images show fluorescence and merged images. Bar = 10 µm.


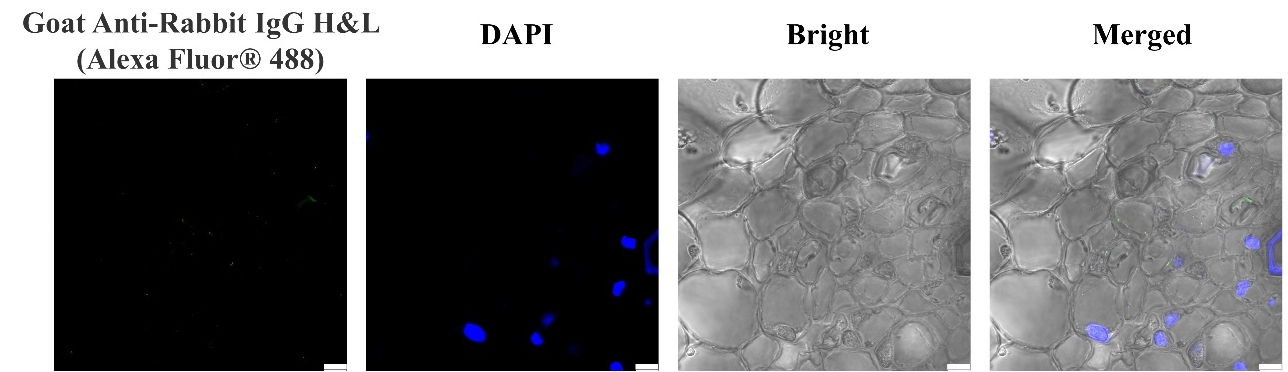


**Fig. S8 Control analyses with affinity-purified preimmune serum.** DAPI-stained nuclei are visible in neighboring phloem cells. Bar = 50 μm


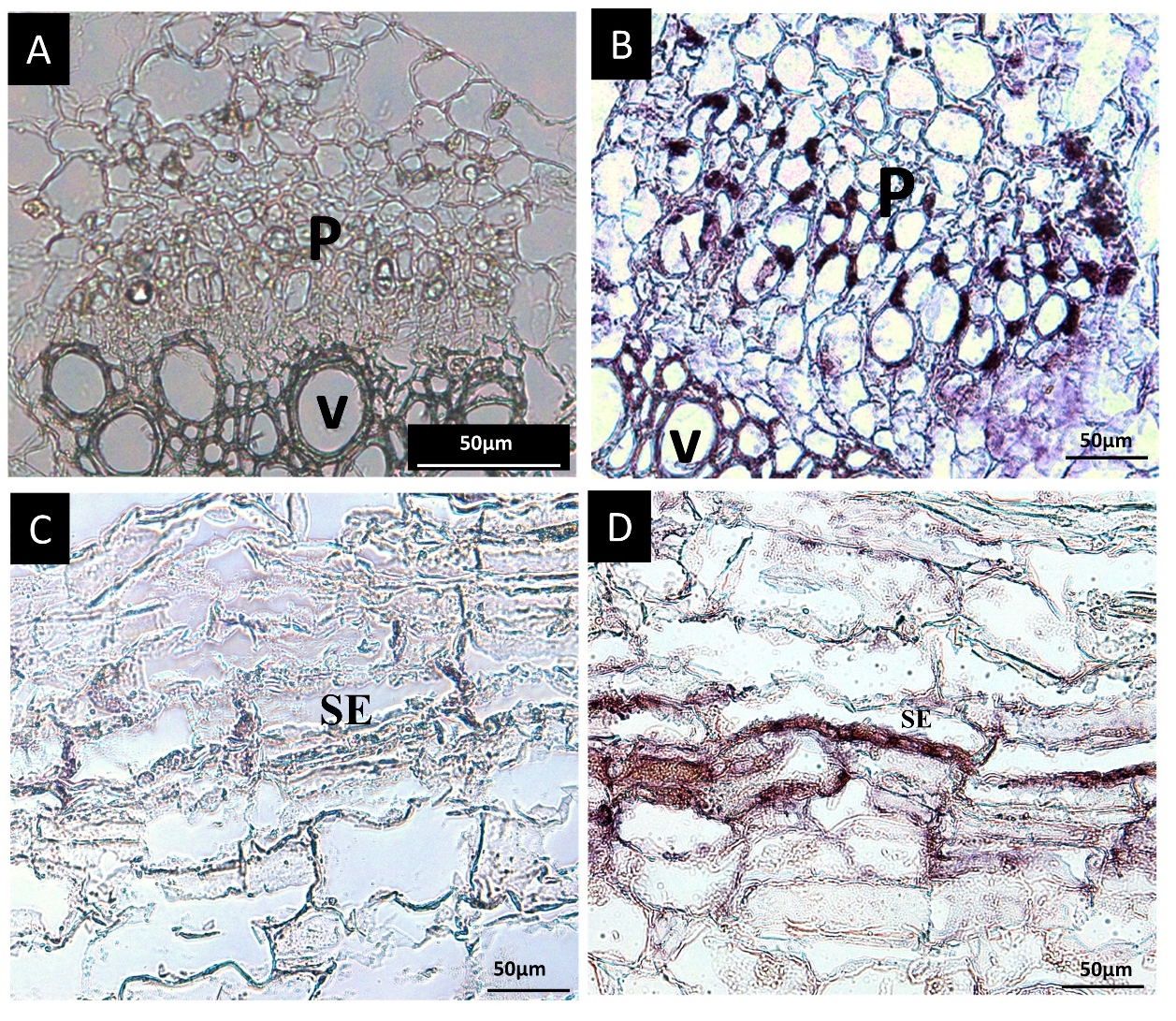


**Fig. S9 Immunohistochemical localization of CmSWEET10 in melon fruit with alkaline phosphatase (AP) staining.** a-b, Transverse sections of fruit vascular bundle (cv. XMS); c-d, Longitudinal sections of fruit vascular bundles (cv. XMS). a, c, Control analyses with preimmune serum. X, xylem; P, phloem. Bar = 50 µm
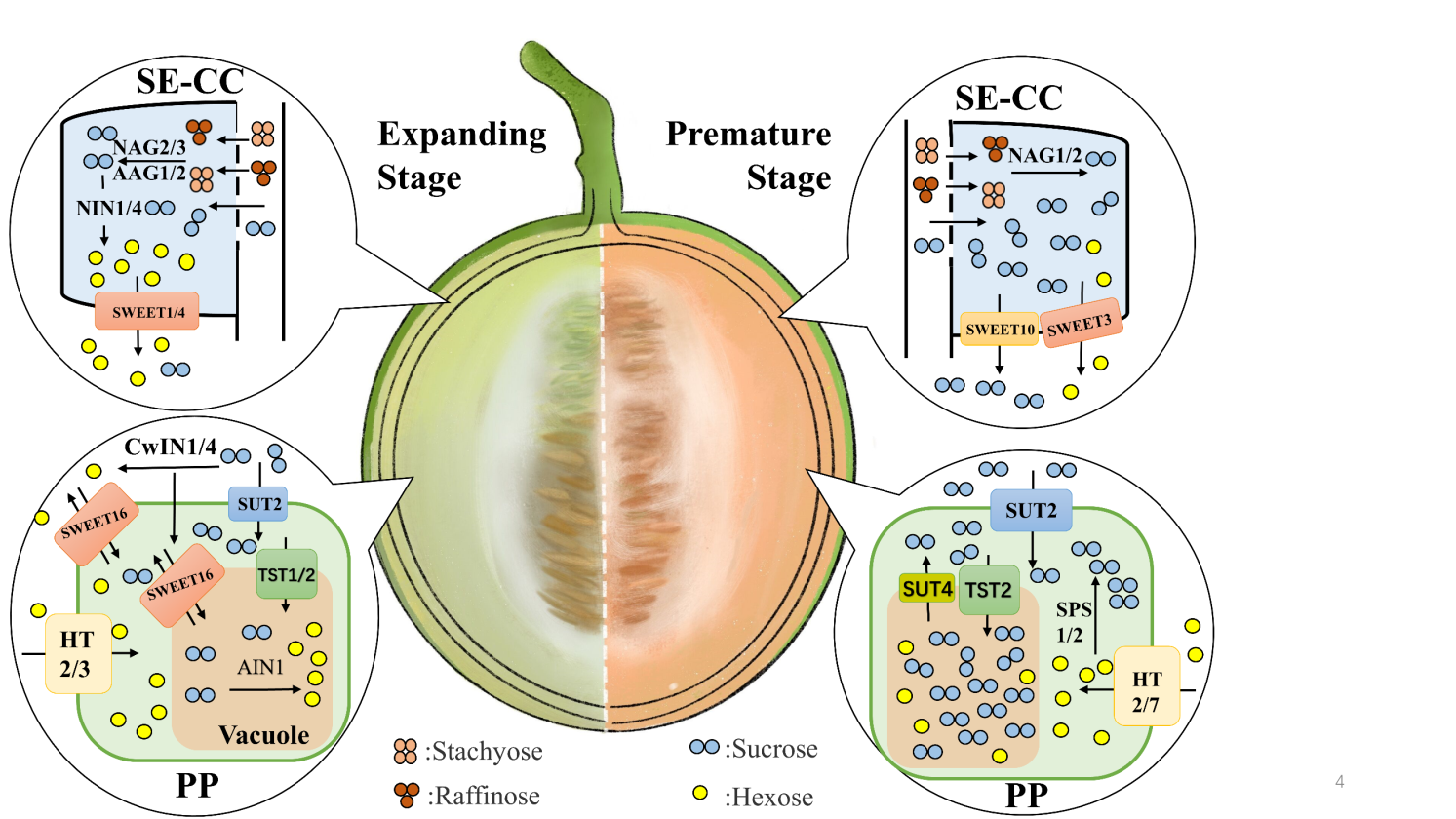


**Fig. S10 Proposed model of flux in sugar metabolism and transport genes expressed in expanding stage (left) and premature stage (right) of melon fruit, as suggested by this study.** Arrows indicate the proposed flux directions at each stage. AAG, acid α-galactosidase. NAG, neutral-alkaline α-galactosidase. SE-CC, sieve element–companion cell. PP, phloem parenchyma.
