# Supplementary material for: Phloem unloading in cultivated melon fruits follows an apoplasmic pathway during enlargement and ripening
Source: Hortic Res. 2023 Jul 4;10(8):uhad123. doi: 10.1093/hr/uhad123 (PMC10405131; doi:10.1093/hr/uhad123)
Supplement: Web_Material_uhad123 [file web_material_uhad123.zip › Supplemental Tables.docx]

Tab.S1 CFDA staining results and phloem unloading patterns of melon fruits. DAP, days after pollination.

| Variety | Classify | Sugar content | | Maturity days | Sampling period（DAP） | Fluorescence concentrated in vascular bundles | Unloading form |
| --- | --- | --- | --- | --- | --- | --- | --- |
| YL | cultivated melon | high | 35 | | >5 | Yes | apoplasmic |
| YN | cultivated melon | high | 35 | | <5 | No | symplastic |
|  |  |  |  |  | ≥5 | Yes | apoplasmic |
| XMS | cultivated agrestis | high | 30 | | ≤5 | No | symplastic |
|  |  |  |  |  | >5 | Yes | apoplasmic |
| YJS | cultivated agrestis | high | 30 | | <5 | No | symplastic |
|  |  |  |  |  | >5 | Yes | apoplasmic |
| M43 | cultivated agrestis | high | 30 | | <5 | No | symplastic |
|  |  |  |  |  | >5 | Yes | apoplasmic |
| BCG | cultivated agrestis | low | 30 | | ≤5 | No | symplastic |
|  |  |  |  |  | >5 | Yes | apoplasmic |
| JKY | wild agrestis | low | 30 | | 0-30 | No | symplastic |
| MPG | wild agrestis | low | 30 | | 0-30 | No | symplastic |

Tab.S2. Soluble solid contents (SSC) in melon fruits of different genotypes. DAP, days after pollination.

| Variety | Sugar content (20 DAP) | Sugar content (30 DAP) |
| --- | --- | --- |
| BLC | 4.96±0.16 | 4.73±0.33 |
| HP | 4.53±0.04 | 4.33±0.24 |
| BCG | 3.33±0.26 | 4.26±0.18 |
| M43 | 7.66±1.03 | 12.5±0.21 |
| LT | 4.3±0.35 | 10.2±0.75 |
| GS | 5.83±0.12 | 13.66±0.76 |
| XMS | 4.06±0.11 | 12.26±0.41 |
| YL | 5.19±0.1 | 13.52±0.51 |

Tab. S3. Gene accession numbers used in this study.

| Gene families | Gene name | Gene ID |
| --- | --- | --- |
| Acid α-galactosidase | *CmAAG1* | MELO3C011770.2 |
|  | *CmAAG2* | MELO3C011771.2 |
| Vacuolar acid invertase | *CmAIN1* | MELO3C005363.2 |
| Cell wall acid invertase | *CmCwIN1* | MELO3C016877.2 |
|  | *CmCwIN4* | MELO3C009488.2 |
| Hexose transporter | *CmHT1* | MELO3C002553.2 |
|  | *CmHT2* | MELO3C007325.2 |
|  | *CmHT3* | MELO3C017655.2 |
|  | *CmHT7* | MELO3C010257.2 |
|  | *CmHT14* | MELO3C026992.2 |
| Neutral-alkaline α-galactosidase | *CmNAG1* | MELO3C025599.2 |
|  | *CmNAG2* | MELO3C023110.2 |
|  | *CmNAG3* | MELO3C010314.2 |
| Neutral invertase | *CmNIN1* | MELO3C024083.2 |
|  | *CmNIN3* | MELO3C004170.2 |
|  | *CmNIN4* | MELO3C006727.2 |
| Sucrose phosphate synthase | *CmSPS1* | MELO3C010300.2 |
|  | *CmSPS2* | MELO3C020357.2 |
| Sugars will eventually be exported transporters | *CmSWEET1* | MELO3C008417.2 |
|  | *CmSWEET3* | MELO3C005869.2 |
|  | *CmSWEET4* | MELO3C016259.2 |
|  | *CmSWEET10* | MELO3C026184.2 |
|  | *CmSWEET16* | MELO3C027076.2 |
|  | *AtSWEET1* | AT1G21460 |
|  | *AtSWEET2* | AT3G14770 |
|  | *AtSWEET3* | AT5G53190 |
|  | *AtSWEET4* | AT3G28007 |
|  | *AtSWEET5* | AT5G62850 |
|  | *AtSWEET6* | AT1G66770 |
|  | *AtSWEET7* | AT4G10850 |
|  | *AtSWEET8* | AT5G40260 |
|  | *AtSWEET9* | AT2G39060 |
|  | *AtSWEET10* | AT5G50790 |
|  | *AtSWEET11* | AT3G48740 |
|  | *AtSWEET12* | AT5G23660 |
|  | *AtSWEET13* | AT5G50800 |
|  | *AtSWEET14* | AT4G25010 |
|  | *AtSWEET15* | AT5G13170 |
|  | *AtSWEET16* | AT3G16690 |
|  | *AtSWEET17* | AT4G15920 |
| Tonoplast sugar transporters | *CmTST1* | MELO3C026522.2 |
|  | *CmTST2* | MELO3C013489.2 |
| Sucrose transporter | *CmSUT2* | MELO3C002966.2 |
|  | *CmSUT4* | MELO3C011115.2 |

Table S4. Primers used in this study.

| primers | Description | Sequence (5’- -3’) |
| --- | --- | --- |
| CmAAG1-qF | Quantitative RT-PCR | GGCTGAGATAGCTCGTGAT |
| CmAAG2-qR | Quantitative RT-PCR | GACCCTTTGCGTGAACATA |
| CmAAG2-qF | Quantitative RT-PCR | ACAATGGTCTCGCTCTAACTC |
| CmAAG2-qF | Quantitative RT-PCR | TCAGCCCAACAATCATCTAA |
| CmAIN1-qF | Quantitative RT-PCR | CTCCTCGTACCTGGCTCTG |
| CmAIN2-qR | Quantitative RT-PCR | CCAAATGGTCCTAAACTACTTCT |
| CmHT1-qF | Quantitative RT-PCR | AAGGCTGTTTGGATGCTTATTC |
| CmHT1-qR | Quantitative RT-PCR | GGGCTCCTCTGTATTTGTATGG |
| CmHT2-qF | Quantitative RT-PCR | TCCGGCCTTAATAATCACC |
| CmHT2-qR | Quantitative RT-PCR | CTTCAACTCCACGTACCCTTCT |
| CmHT3-qF | Quantitative RT-PCR | CTCTTCTCCGGCCTCGTTT |
| CmHT3-qR | Quantitative RT-PCR | TAGCTCCTCTGTAGTTTGATGGTG |
| CmHT7-qF | Quantitative RT-PCR | GGTGGTCGCCATTATCCT |
| CmHT7-qR | Quantitative RT-PCR | CACTGTGATGCTTTGTCCC |
| CmHT14-qF | Quantitative RT-PCR | TAGCAACGAGGCACAAGC |
| CmHT14-qR | Quantitative RT-PCR | GGAACATTACAGGAGCATAGAAC |
| CmNAG1-qF | Quantitative RT-PCR | TTTGGTGTTTGTGGGTGC |
| CmNAG1-qR | Quantitative RT-PCR | TGACGCCATCTGAAGTGA |
| CmNAG2-qF | Quantitative RT-PCR | CACGGGAAGACCATACTGA |
| CmNAG2-qR | Quantitative RT-PCR | ACCATAACTTGAAACGGAAAC |
| CmNAG3-qF | Quantitative RT-PCR | TCGCATACGATTCACATAGC |
| CmNAG3-qR | Quantitative RT-PCR | CCGGTGCATCACTGACATAAA |
| CmNIN1-qF | Quantitative RT-PCR | TGATACGTTCCCAACTTTACTT |
| CmNIN1-qR | Quantitative RT-PCR | ACGCTTCACAATACGCTCT |
| CmNIN3-qF | Quantitative RT-PCR | GCAATCCCGACTCTATCAA |
| CmNIN3-qR | Quantitative RT-PCR | ACTTCTTCCTGCCCGTCT |
| CmNIN4-qF | Quantitative RT-PCR | AAATTCCTAGTTGGCTGGTT |
| CmNIN4-qR | Quantitative RT-PCR | AGATTCCCGAGCGTAAA |
| CmSPS1-qF | Quantitative RT-PCR | TGTTTGTCGGAGAAAGTGG |
| CmSPS1-qR | Quantitative RT-PCR | CGTCGGAAAGAGGGTAGTT |
| CmSPS2-qF | Quantitative RT-PCR | GGGACGAAATATCCACCG |
| CmSPS2-qR | Quantitative RT-PCR | TGACTCATTCCCTGACCTCTT |
| CmSWEET1-qF | Quantitative RT-PCR | CTATTGCTATGTATGCCTCT |
| CmSWEET1-qR | Quantitative RT-PCR | AACTTGCGGAGAATGAGA |
| CmSWEET3-qF | Quantitative RT-PCR | CTATTGCTATGTATGCCTCT |
| CmSWEET3-qR | Quantitative RT-PCR | AACTTGCGGAGAATGAGA |
| CmSWEET4-qF | Quantitative RT-PCR | GTTCTTCTCGTGCTACTCATCG |
| CmSWEET4-qF | Quantitative RT-PCR | GAGCATAAATAGTCCAAGCCAC |
| CmSWEET10-qR | Quantitative RT-PCR | GGTTCGGGTTGATGCTGG |
| CmSWEET10-qF | Quantitative RT-PCR | CACGCTCTTCGTCTTTATCACTT |
| CmSWEET16-qF | Quantitative RT-PCR | TTGGTCTGCCTATGCTGTTC |
| CmSWEET16-qR | Quantitative RT-PCR | TCCTCCATCATCTCCGTTG |
| CmTST1-qF | Quantitative RT-PCR | ATAATAGGTCCTGCTGAAGA |
| CmTST1-qR | Quantitative RT-PCR | AAAGGGTGACAAGTGGGT |
| CmTST2-qF | Quantitative RT-PCR | GGATGGGATAATGCGACTA |
| CmTST2-qR | Quantitative RT-PCR | GCTCCGATAAGGGATGTG |
| CmSUT2-qF | Quantitative RT-PCR | TAAGACATTTACCACCTGC |
| CmSUT2-qR | Quantitative RT-PCR | TACACTTCCCTTCCCATC |
| CmSUT4-qF | Quantitative RT-PCR | GGTTCCACTGAGACGGTTAC |
| CmSUT4-qR | Quantitative RT-PCR | ATCGGTCCACAGAGCCATA |
| Tubulin F | Quantitative RT-PCR | GTTCTTATCAATGCTGGTGGTG |
| Tubulin R | Quantitative RT-PCR | TTTACTCACAGTCCCTTGGTCTC |
| SWEET1-196F | Yeast uptake | atcgataccgtcgacctcgagATGGATATTCCGCACTTCTTGTT |
| SWEET1-196R | Yeast uptake | ggtaccgggccccccctcgagTTAAACTTGATCATCACGGTCCACT |
| SWEET10-196F | Yeast uptake | atcgataccgtcgacctcgagATGGCCATCAGTCCCCAAACTCTT |
| SWEET10-196R | Yeast uptake | ggtaccgggccccccctcgagTTAGTTAGTTATGATGTCTTGGTCT |
| SWEET1-GFPF | Translational fusion to GFP | attacgccgaggtcATGGATATTCCGCACTTCTTGTT |
| SWEET1-GFPR | Translational fusion to GFP | tagggaagaggATGGATATTCCGCACTTCTTGTT |
| SWEET10-GFPF | Translational fusion to GFP | attacgccgaggtcATGGCCATCAGTCCCCAAACTCTT |
| SWEET10-GFPR | Translational fusion to GFP | tagggaagaggGTTAGTTATGATGTCTTGGTCT |
| SWEET16-GFPF | Translational fusion to GFP | attacgccgaggtcATGAAGATGGTTGGTTTATTCAATG |
| SWEET16-GFPR | Translational fusion to GFP | tagggaagaggTCAAAATTTTCCATCTTCCACCTCA |
